# Data on the effect of temperature variation tendency on the inhibitive absorption of *Lasienthera africanum* in 0.5M HCl: A necessity

**DOI:** 10.1016/j.dib.2018.09.019

**Published:** 2018-09-12

**Authors:** J. Fayomi, A.P.I. Popoola, O.S.I. Fayomi, K.O. Babaremu

**Affiliations:** a Department of Chemical, Metallurgical and Materials Engineering, Tshwane University of Technology, P.M.B. X680, Pretoria South Africa; b Department of Mechanical Engineering, Covenant University, P.M.B 1023 Ota Nigeria

**Keywords:** Weight loss, Temperature, HCl solution, *Lasienthera africanum*

## Abstract

The assessment of *Lasienthera africanum* as corrosion inhibitor for aluminium alloy in 0.5M HCl acid solution using weight loss method was investigated at 303 and 313 K to check its behaviour at high temperature application. Inhibitor efficiency (IE) as high as 93.8, 87.3% both at 303 and 313 K, respectively, was obtained. It can be seen that the inhibition efficiency values increase with increase in extract concentration which suggests that the inhibition is due to the adsorption of the inhibitor on the metal surface. A straight line is obtained when *C*/*θ* is plotted against *C* with linear correlation coefficient of the fitted data close to 1. The adsorption of the inhibitor molecules obey Lanqmuir׳s adsorption isotherm.

**Table:** 

Subject area	*Material science*
More specific subject area	*Corrosion science and engineering*
Type of data	*Table, graph*
How data was acquired	The data in this work was acquired by weight loss method with variation in temperature.
Data format	Raw, Analysed.
Experimental factors	The aluminium coupons were properly weighed before and after immersion into the test solution. The inhibitor was test against temperature variation of 303 K and 313 K.
Experimental features	The immersions were performed between 60–300 min at a temperature of 303 K and 313 K. The effect of inhibitor on the properties of aluminium alloy was acquired. The framework of temperature variation condition as it influences the corrosion rate and efficiency was properly observed.
Data source location	Department of Chemical, Metallurgical and Materials Engineering, Tshwane University of Technology, Pretoria, South Africa and Mechanical Engineering, Covenant University, Ota Ogun State, Nigeria.
Data accessibility	Data are available within this article
Related research article	*n/a*

**Value of the data**

• The given data will show author in the field of corrosion science the effect of 0.5 HCl concentration on aluminium corrosion, with or with out inhibitor.
• The data obtained could be used to check the correlation of temperature variation on the corrosion of aluminum coupon in acidic medium.
• The data could be used to check the effect of increase in temperature on the inhibition efficiency of the inhibitor used (*lasienthera africanum)*
• The results obtained shows that the inhibition potency of the inhibitor decreases with time in the contaminated environment.

## 1 Data

The weight losses with depth of immersion were collected and a unique set of experimental frame work data were generated. The depositions process was performed between 60–300 at a varying temperature of 303 k and 313 k. The data acquired from the weight loss measurements of the aluminum coupon is presented in Table 1, Table 2, Table 3, Table 4, Table 5, Table 6, Table 7, Table 8, Table 9, Table 10, Table 11, Table 12, Table 13, Table 14 below. From the weight loss result, the corrosion rates were calculated and the inhibitor efficiency obtained. The data obtained shows that the rate of corrosion decreases with increase in the concentration of inhibitors, this is also true for the varying temperature though with increase in time the inhibitor efficiency decreases.

**Table 1:** Aluminum coupon in 0.5M HCl at 30 °C without *Lasienthera africanum* extract (control 1).

**Time (Min)**	**Initial weight of specimen, W**_**1**_	**Final Weight Of Specimen, W**_**f**_	**Weight Loss, ∆W**_**(g)**_	**Inhibition efficiency (%)**	**Corrosion rate (mm/yr.)**
60	1.0068	1.0036	0.0032	–	8.65
120	1.0068	0.9979	0.0089	–	12.03
180	1.0068	0.9858	0.0210	–	18.93
240	1.0068	0.9732	0.0336	–	22.71
300	1.0068	0.9695	0.0373	–	20.17

**Table 2:** Aluminum coupon in 0.5M HCl at 30 °C without *Lasienthera africanum* extract (control 2).

**Time (Min)**	**Initial weight of specimen, W**_**1**_	**Final Weight Of Specimen, W**_**f**_	**Weight Loss, ∆W**_**(g)**_	**Inhibition efficiency (%)**	**Corrosion rate (mm/yr.)**
60	0.9655	0.9623	0.0032	–	8.65
120	0.9655	0.9573	0.0082	–	11.09
180	0.9655	0.9523	0.0132	–	11.90
240	0.9655	0.9491	0.0164	–	11.09
300	0.9655	0.09435	0.0220	–	11.90

**Table 3:** Aluminum coupon in 0.5M HCl at 30 °C containing 10 mg/l *Lasienthera africanum* extract.

**Time (Min)**	**Initial weight of specimen, W**_**1**_	**Final weight of specimen, W**_**f**_	**Weight loss, ∆W**_**(g)**_	**Inhibition efficiency (%)**	**Corrosion rate (mm/yr.)**
60	1.0101	1.0094	0.0007	78.1	1.89
120	1.0101	1.0038	0.0063	26.7	8.52
180	1.0101	0.9966	0.0135	21.1	12.17
240	1.0101	0.9873	0.0228	8.8	15.41
300	1.0101	0.9821	0.0280	5.7	15.14

**Table 4:** Aluminum coupon in 0.5M HCl at 30 °C containing 20 mg/l *Lasienthera africanum* extract.

**Time (Min)**	**Initial weight of specimen, W**_**1**_	**Final weight of specimen, W**_**f**_	**Weight loss, ∆W**_**(g)**_	**Inhibition efficiency (%)**	**Corrosion rate (mm/yr.)**
60	1.0170	1.0165	0.0005	84.4	1.35
120	1.0170	1.0113	0.0057	33.7	7.71
180	1.0170	1.0047	0.0123	28.1	11.09
240	1.0170	0.9982	0.0188	24.8	12.71
300	1.0170	0.9910	0.0260	12.5	14.06

**Table 5:** Aluminum coupon in 0.5M HCl at 30 °C containing 40 mg/l *Lasienthera africanum* extract.

**Time (Min)**	**Initial weight of specimen, W**_**1**_	**Final weight of specimen, W**_**f**_	**Weight loss, ∆W**_**(g)**_	**Inhibition efficiency (%)**	**Corrosion rate (mm/yr.)**
60	1.0580	1.0576	0.0004	87.5	1.08
120	1.0580	1.0540	0.0040	53.5	5.48
180	1.0580	1.0491	0.0089	47.9	8.02
240	1.0580	1.0423	0.0157	37.2	10.61
300	1.0580	1.0324	0.0256	14.5	13.84

**Table 6:** Aluminum coupon in 0.5M HCl at 30 °C containing 60 mg/l *Lasienthera africanum* extract.

**Time (Min)**	**Initial weight of specimen, W**_**1**_	**Final weight of specimen, W**_**f**_	**Weight loss, ∆W**_**(g)**_	**Inhibition efficiency (%)**	**Corrosion rate (mm/yr.)**
60	0.9900	0.9897	0.0003	90.6	0.81
120	0.9900	0.9876	0.0024	72.1	3.24
180	0.9900	0.9849	0.0051	70.1	4.60
240	0.9900	0.9754	0.0146	41.6	9.86
300	0.9900	0.96620	0.0238	19.8	11.95

**Table 7:** Aluminum coupon in 0.5M HCl at 30 °C containing 80 mg/l *Lasienthera africanum* extract.

**Time (Min)**	**Initial weight of specimen, W**_**1**_	**Final weight of specimen, W**_**f**_	**Weight loss, ∆W**_**(g)**_	**Inhibition efficiency (%)**	**Corrosion rate (mm/yr.)**
60	0.9824	0.9822	0.0002	93.8	0.54
120	0.9824	0.9803	0.0021	75.6	2.84
180	0.9824	0.9776	0.0048	71.9	4.33
240	0.9824	0.9694	0.0130	48.0	8.79
300	0.9824	0.9603	0.0221	25.6	11.95

**Table 8:** Aluminum coupon in 0.5M HCl at 40 °C without *Lasienthera africanum* extract (control 1).

**Time (Min)**	**Initial weight of specimen, W**_**1**_	**Final weight of specimen, W**_**f**_	**Weight loss, ∆W**_**(g)**_	**Inhibition efficiency (%)**	**Corrosion rate (mm/yr.)**
60	1.0556	1.0242	0.0314	–	84.90
120	1.0556	1.0008	0.0548	–	74.08
180	1.0556	0.9949	0.0607	–	54.70
240	1.0556	0.9849	0.0707	–	47.79
300	1.0556	0.9563	O.0993	–	53.70

**Table 9:** Aluminum coupon in 0.5M HCl at 40 °C without *Lasienthera africanum* extract (control 2).

**Time (Min)**	**Initial weight of specimen, W**_**1**_	**Final weight of specimen, W**_**f**_	**Weight loss, ∆W**_**(g)**_	**Inhibition efficiency (%)**	**Corrosion rate (mm/yr.)**
60	1.0563	1.0436	0.0127	–	34.34
120	1.0563	1.0258	0.0405	–	54.75
180	1.0563	0.9886	0.0677	–	61.01
240	1.0563	0.9825	0.0731	–	49.41
300	1.0563	0.9669	0.0894	–	48.34

**Table 10:** Aluminum coupon in 0.5M HCl at 40 °C containing 10 mg/l *Lasienthera africanum* extract.

**Time (Min)**	**Initial weight of specimen, W**_**1**_	**Final weight of specimen, W**_**f**_	**Weight loss, ∆W**_**(g)**_	**Inhibition efficiency (%)**	**Corrosion rate (mm/yr.)**
60	1.0247	1.0134	0.0113	48.9	30.55
120	1.0247	0.9905	0.0342	28.3	46.23
180	1.0247	0.9745	0.0502	21.8	45.24
240	1.0247	0.9660	0.0587	18.4	39.68
300	1.0247	0.9392	0.0855	9.3	46.23

**Table 11:** Aluminum coupon in 0.5M HCl at 40 °C containing 20 mg/l *Lasienthera africanum* extract.

**Time (Min)**	**Initial weight of specimen, W**_**1**_	**Final weight of specimen, W**_**f**_	**Weight loss, ∆W**_**(g)**_	**Inhibition efficiency (%)**	**Corrosion rate (mm/yr.)**
60	1.0205	1.0121	0.0084	61.9	22.71
120	1.0205	0.9959	0.0246	48.4	33.25
180	1.0205	0.9845	0.0360	43.9	32.44
240	1.0205	0.9750	0.0450	37.4	30.42
300	1.0205	0.9538	0.0667	29.2	36.07

**Table 12:** Aluminum coupon in 0.5M HCl at 40 °C containing 40 mg/l *Lasienthera africanum* extract.

**Time (Min)**	**Initial weight of specimen, W**_**1**_	**Final weight of specimen, W**_**f**_	**Weight loss, ∆W**_**(g)**_	**Inhibition efficiency (%)**	**Corrosion rate (mm/yr.)**
60	1.3374	1.3306	0.0068	69.2	18.39
120	1.3374	1.3214	0.0160	66.5	21.63
180	1.3374	1.3111	0.0263	59.0	23.70
240	1.3374	1.2957	0.0417	42.0	28.19
300	1.3374	1.2803	0.0571	39.4	30.88

**Table 13:** Aluminum coupon in 0.5M HCl at 40 °C containing 60 mg/l *Lasienthera africanum* extract.

**Time (Min)**	**Initial weight of specimen, W**_**1**_	**Final weight of specimen, W**_**f**_	**Weight loss, ∆W**_**(g)**_	**Inhibition efficiency (%)**	**Corrosion rate (mm/yr.)**
60	1.0130	1.0084	0.0046	79.2	12.44
120	1.0130	1.0020	0.0110	76.9	14.87
180	1.0130	0.9945	0.0185	71.2	16.67
240	1.0130	0.9785	0.0345	52.0	23.32
300	1.0130	0.9585	0.0545	42.2	29.47

**Table 14:** Aluminum coupon in 0.5M HCl at 40 °C containing 80 mg/l *Lasienthera africanum* extract.

**Time (Min)**	**Initial weight of specimen, W**_**1**_	**Final weight of specimen, W**_**f**_	**Weight loss, ∆W**_**(g)**_	**Inhibition efficiency (%)**	**Corrosion rate (mm/yr.)**
60	0.9478	0.9450	0.0028	87.3	7.57
120	0.9478	0.9394	0.0084	84.2	11.36
180	0.9478	0.9349	0.0129	79.9	11.63
240	0.9478	0.9230	0.0248	65.5	16.76
300	0.9478	0.9120	0.0356	62.2	19.25

## 2 Experimental design, materials, and methods

The materials employed in this work include Aluminum coupons, Thermometer, Analytical weighing balance, heating mantle, Magnetic stirrer, water bath, dessicator.

### 2.1 Reagents

Hydrochloric acid (HCl), Ethanol, Acetone,

### 2.2 Plant sample

*Lasienthera africanum* extract.

### 2.3 Preparation of aluminum coupons for anti-corrosion study

Aluminum sheets of purity 98.8% were used in this study each sheet was 0.14 cm thick and was mechanically cut into rectangular coupons of dimension 3 cm × 4 cm. The total surface area of the coupon used was 12 cm^2^. These coupons were further polished, degreased in ethanol and dried in acetone (Ita and Edem, 2000). The coupons were then stored in a moisture-free desiccator to avoid contamination before using them for corrosion studies. The initial weight of each sample was taken and recorded. All reagents used were of analytical grade. They were used as sourced with no further purification. An aqueous solution of 0.5M was used as blank solution.

### 2.4 Preparation of plant extract for corrosion inhibition studies

*Lasienthera africanum* leaf sample was bought from Effurun market in Delta state Nigeria and was properly washed. The sample was further dried and ground into fine powder. The acidic leaf extract was prepared by adding 5.0 g of the plant sample in 100 ml 0.5M. The resulting solution was boiled for 3 h and allowed to stand before filtering. It was observed that 0.1541 g of organic soluble matter dissolved in the acidic medium. This becomes the stock solution and from these, concentrations of 10 mg/l, 20 mg/l, 40 mg/l, 60 mg/l, 80 mg/l were made.

### 2.5 Weight loss measurement

This work involved the introduction of already prepared concentrations of the inhibitor into separate beakers maintained at room temperature. A total of seven beakers label (A–E and X and Y) were used; with A, B, C, D, E, containing 100 ml of the acidic extract solution while X and Y were used as the blank (control) for the experiment. The beakers label A to E contains different volumes of stock solution of the inhibitor with concentrations of 10 mg/l, 20 mg/l, 40 mg/l, 60 mg/l, 80 mg/l which was made up to 100 ml each into different concentration of 0.5M HCl.

Previously weighed aluminum coupons were then placed in the test solution. Each coupon was retrieved from the test solutions progressively for 1 h in total of 5 h. After the immersion test, the specimens were carefully dip in water and then properly cleansed to remove loose segments of the film of the corroded samples following by degreasing in ethanol and drying with acetone. The difference in weight of the coupons was again taken as the weight loss. From the initial weight of the aluminum coupons, the weight loss, the corrosion rate (CR) and inhibition efficiency were determined.

## 3 Results analysis

**Fig. 1:**
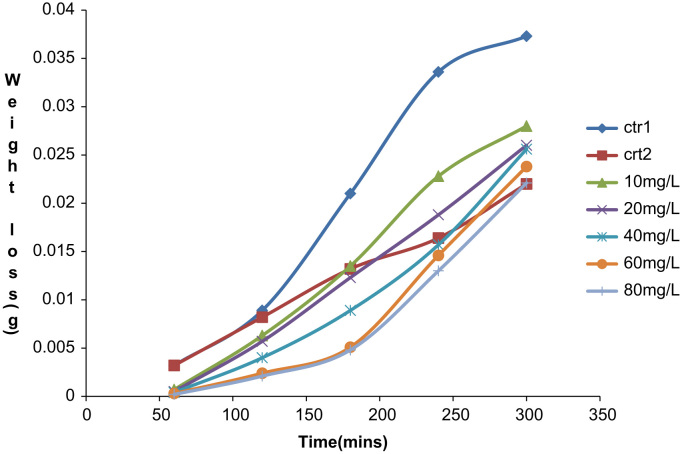
Plot of weight loss against time at 30 °C in 0.5M HCl.

**Fig. 2:**
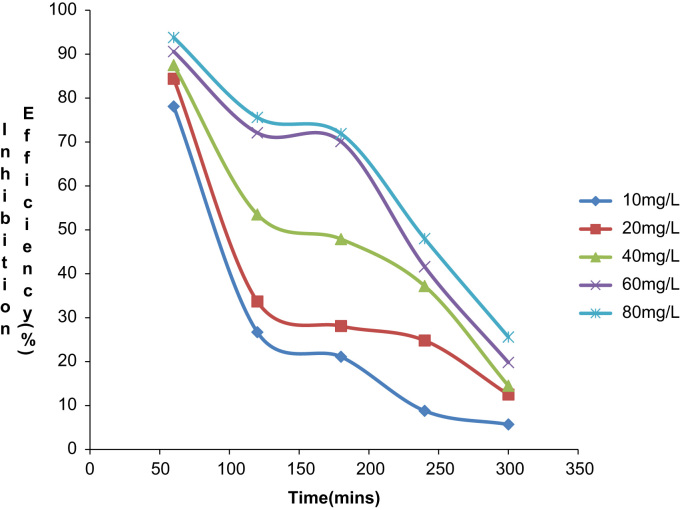
Plot of Inhibition efficiency against time at 30 °C in 0.5M HCl.

**Fig. 3:**
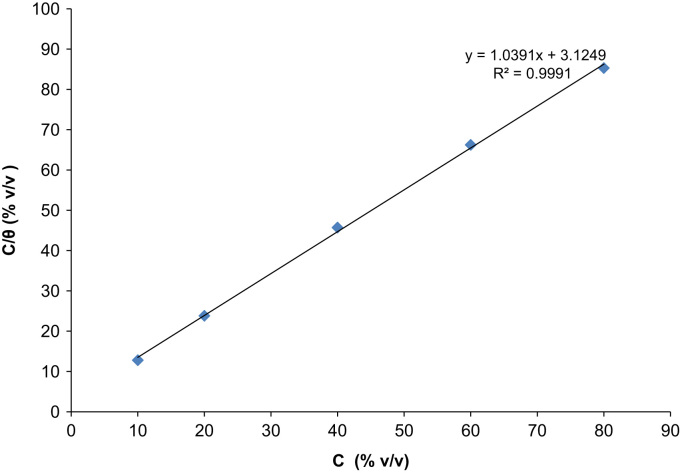
Langmuir adsorption model on the aluminum surface of *Lasienthera africanum* extract in 0.5M HCl solution for 60 min immersion period at 30 °C.

**Fig. 4:**
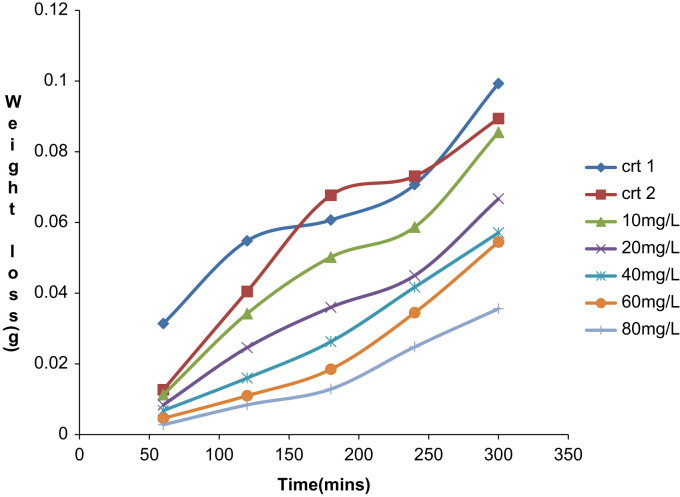
Plot of weight loss against time at 40 °C IN 0.5M HCl.

**Fig. 5:**
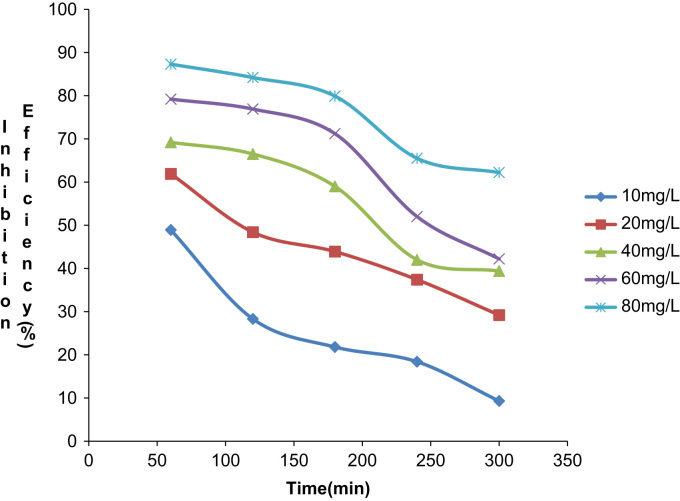
Plot of inhibition efficiency against time at 40 °C in 0.5M HCl.

**Fig. 6:**
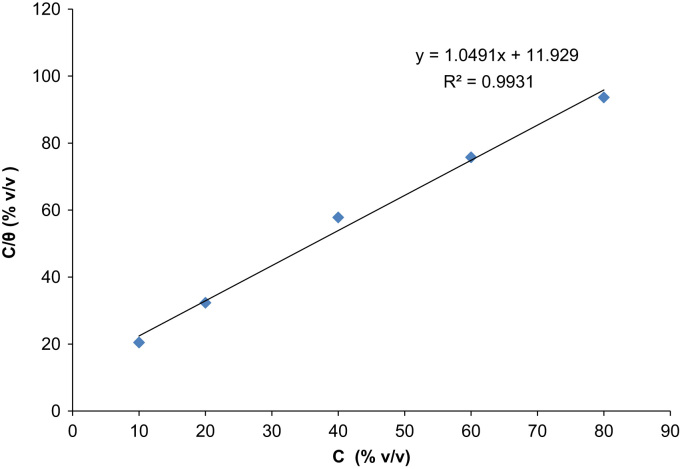
Langmuir adsorption model on the aluminum surface of *Lasienthera africanum* extract in 0.5M HCl solution for 60 min immersion period at 40 °C.

The depositions process was performed between 60 min and 300 min at a varying temperature of 303 K and 313 K. The data acquired from the weight loss measurements of the aluminum coupon is presented in Table 1, Table 2, Table 3, Table 4, Table 5, Table 6, Table 7, Table 8, Table 9, Table 10, Table 11, Table 12, Table 13, Table 14 above. From the weight loss above, the corrosion rates were calculated and the inhibitor efficiency obtained. As presented in Table 1, Table 2, Table 3, Table 4, Table 5, Table 6, Table 7, Table 8, Table 9, Table 10, Table 11, Table 12, Table 13, Table 14, the percentage inhibition efficiency values increase with increasing extract concentration and the highest inhibition efficiency value of 93.8 and 87.3% was obtained at 80 mg/l concentration at 303 K and 313 K, respectively. A straight line is obtained when *C*/*θ* is plotted against *C* with linear correlation coefficient of the fitted data close to 1. The adsorption of the inhibitor molecules obey Lanqmuir׳s adsorption isotherm expressed as equation below (Fig. 1, Fig. 2, Fig. 3, Fig. 4, Fig. 5, Fig. 6).

C/θ=C+1/K

Where *C* is the inhibitor concentration and *K* the equilibrium constant for the adsorption/desorption process of the inhibitor molecules on the metal surface.

