# Supplementary material for: Data on the effect of temperature variation tendency on the inhibitive absorption of Lasienthera africanum in 0.5M HCl: A necessity
Source: Data Brief. 2018 Sep 12;20:2003–11. doi: 10.1016/j.dib.2018.09.019 (PMC6172414; doi:10.1016/j.dib.2018.09.019)
Supplement: Supplementary file 1 — Supplementary material [file mmc1.docx]

***COVER LETTER/CONFLICT OF INTEREST ATTESTATION***

*30^th^ August, 2018*

*The Editor-in-Chief*

*Data in Brief*

***Subject:***  ***NO CONFLICT OF INTEREST***

*Dear Sir,*

*This serve to notify you that the manuscript is original of the authors work and there is no conflict of interest of any kind regarding the manuscript Data on the effect of Temperature Variation Tendency on the Inhibitive Absorption of Lasienthera Africanum in 0.5M HCl: A Neccesity*

*Sincerely yours,*

*Juwon Fayomi*

*Department of Chemical, Metallurgical and Materials Engineering, Tshwane University of Technology, P.M.B. X680, Pretoria, South Africa.*
